# Cycling hypoxia promotes a pro-inflammatory phenotype in macrophages via JNK/p65 signaling pathway

**DOI:** 10.1038/s41598-020-57677-5

**Published:** 2020-01-21

**Authors:** Victor Delprat, Céline Tellier, Catherine Demazy, Martine Raes, Olivier Feron, Carine Michiels

**Affiliations:** 1 Unit of Biochemistry and Cellular Biology (URBC), Namur Research Institute for LIfe Sciences (NARILIS), University of Namur (UNamur), 61 Rue de Bruxelles, B-5000 Namur, Belgium; 2 Pole of Pharmacology and Therapeutics (FATH 5349), Institut de recherche expérimentale et clinique, UCLouvain, 57 Avenue Hippocrate, B-1200 Brussels, Belgium

**Keywords:** Cancer microenvironment, Oncology

## Abstract

Cycling hypoxia (cyH), also called intermittent hypoxia, occurs in solid tumors and affects different cell types in the tumor microenvironment and in particular the tumor-associated macrophages (TAMs). As cyH and TAMs both favor tumor progression, we investigated whether cyH could drive the pro-tumoral phenotype of macrophages. Here, the effects of cyH on human THP-1 macrophages and murine bone marrow-derived macrophages (BMDM), either unpolarized M0, or polarized in M1 or M2 phenotype were studied. In M0 macrophages, cyH induced a pro-inflammatory phenotype characterized by an increase in TNFα and IL-8/MIP-2 secretion. CyH amplified the pro-inflammatory phenotype of M1 macrophages evidenced by an increased pro-inflammatory cytokine secretion and pro-inflammatory gene expression. Furthermore, cyH increased c-jun activation in human M0 macrophages and highly increased c-jun and NF-κB activation in M1 macrophages. C-jun and p65 are implicated in the effects of cyH on M0 and M1 macrophages since inhibition of their activation prevented the cyH pro-inflammatory effects. In conclusion, we demonstrated that cyH induces or amplifies a pro-inflammatory phenotype in M0 and M1 macrophages by activating JNK/p65 signaling pathway. These results highlight a specific role of cyH in the amplification of tumor-related inflammation by modulating the inflammatory phenotype of macrophages.

## Introduction

Tumors are complex tissues composed of multiple cell types interacting and influencing each other, namely malignant cells and stromal cells like endothelial cells, immune cells and fibroblasts^1^. The intricate combination of tumor microenvironment composition and environmental factors strongly determines tumor outcome^2^. A major factor altering the tumor microenvironment is the presence of low oxygen tension called hypoxia, that is a common feature of malignant tumors^3^. Two types of hypoxia can be distinguished: chronic and cycling hypoxia (cyH). Chronic hypoxia (chH) is associated to the limited oxygen distribution in a tissue; it is mainly the result of uncontrolled proliferation of O_2_-consuming cancer cells and the O_2_ diffusion gradient from blood capillaries^4,5^. In contrast, cyH, also called intermittent hypoxia, is related to the irregular erythrocyte flux circulating in the anarchical tumor blood network characterized by the presence of temporary occlusions^6–8^. The instability of blood flow leads to periods of hypoxia followed by periods of reoxygenation, occurring over hours through a clear pattern of periodicity^9^. We previously demonstrated that cyH amplifies the endothelial inflammatory response induced by TNFα notably through an overactivation of NF-κB. Moreover, we showed that cyH enhances the overall tumor inflammation characterized by a global increase in inflammatory gene expression and by an increase in intratumor leukocyte infiltration in tumor-bearing mice^10^. Inflammation is indeed described to be mutagenic and to favor proliferation and survival of malignant cells, angiogenesis, metastasis, corruption of the adaptive immune system and resistance to treatments^11^. Tumor-promoting inflammation has been designated as a new enabling characteristic for cancer, contributing to the acquisition of multiple hallmark capabilities^12^. Inflammation is firstly designed to fight disorders like infections or transformed cells. In a normal tissue, inflammation is resolved when disorders are eliminated. However, for malignant tumors that evade immune system, chronic inflammation persists^13^.

All the cell types present in the tumor microenvironment could participate in the tumor-related inflammation but a major role is assigned to immune cells^12^. Macrophages constitute the main leukocytic infiltrate in tumors and are referred as tumor-associated macrophages (TAMs). Their extensive infiltration correlates with poor patient prognosis in more than 80% of analyzed cancers^14^. As these cells display a remarkable plasticity, their phenotype highly varies in function of environmental cues. Macrophages have been classified along a continuum of functional states where M1 and M2 are the two extreme polarization phenotypes^15,16^. M1 macrophage polarization refers to the classical activation in response to TLR ligands (such as LPS) and IFNγ whereas M2 polarization constitutes an alternative activation of macrophages induced by IL-4 and/or IL-13^17^. M1 macrophages are pro-inflammatory as they are characterized by the secretion of high amounts of pro-inflammatory cytokines (e.g. TNFα, IL-1β, IL-6). They play major roles in host defense by phagocytosing and killing pathogens, by releasing cytotoxic components like NO and by activating the adaptive immune system through antigen presentation and T cell activation. M2 macrophages are anti-inflammatory characterized by the secretion of anti-inflammatory cytokines (e.g. CCL22, IL-10, CCL-18). They induce the resolution of inflammation (e.g. via an increased expression of MRC-1 also named CD206) and tissue repair (e.g. via the increased production of fibronectin^18^). By receiving particular local signals, different subpopulations of macrophages reside in the same tumor, changing according to their localization and along the time of tumor progression^19,20^.

We postulated that cyH could modulate macrophage phenotype towards a phenotype that could promote tumor inflammation. In this study, we investigated the effects of cyH and chH on the polarization of human THP-1 macrophages and murine bone marrow-derived macrophages (BMDM). Results showed that cyH induces, on its own, a pro-inflammatory phenotype in unpolarized human and murine macrophages and reinforces the pro-inflammatory phenotype of human and murine M1 macrophages through the activation of the JNK/p65 signaling pathway.

## Material and Methods

### Cell culture and hypoxia incubation

Human monocytic THP-1 cells were maintained, until 12–13 passages, in RPMI medium 1640 with L-glutamine (#11875-093, Gibco) supplemented with 10 mM HEPES (#15630-056, Gibco), 1 mM pyruvate (#11360-039, Gibco), 0.05 mM β-mercaptoethanol (#31350-010, Gibco) and 2.5 g/l D-glucose (Merck), and containing 10% of heat decomplemented FBS. THP-1 monocytes were seeded at 800,000 cells/well in 6-well plates (Costar) and directly differentiated into macrophages by 24 h incubation with 150 nM phorbol 12-myristate 13-acetate (PMA, # P8139, Sigma) followed by 24 h rest period in complete RPMI medium without PMA. At the end of 48 h, THP-1 macrophages were used as M0 macrophages or were polarized into M1 or M2 macrophages. For M1 polarization, macrophages were incubated for 24 h with 10 pg/ml LPS (#L8630, Sigma) and 20 ng/ml rhIFNγ (R&D Systems). For M2 polarization, macrophages were incubated for 48 h with 20 ng/ml rhIL-4 (R&D Systems) and 20 ng/ml rhIL-13 (R&D Systems).

Murine bone marrow-derived macrophages (BMDM) were obtained from the differentiation of monocytes recovered from femur and tibia bone marrow of male C57BL6 mice, 6–8 weeks old. The local ethic committee of the university of Namur (Commission d’éthique en expérimentation animale; CEEXPANI) approved the procedure according to the animal care regulation (agreement number 14 229, University of Namur). All experiments were performed in accordance with their relevant guidelines and regulations. Bone marrow cells were firstly transferred in 100 mm dishes in DMEM high glucose containing 4.5 g/l D-glucose, L-glutamine and sodium pyruvate (#11995, Gibco) + 10% heat decomplemented low endotoxin FBS (HIS-LE FBS, #F7524, Sigma). 24 h later, non-adhering cells comprising monocytes were harvested and then monocyte differentiation into macrophages was launched for 6 days by adding 10% of conditioned media of L-929 mouse fibroblasts (L-929 CM), enriched in M-CSF. Conditioned medium was generated by seeding 500,000 L-929 cells in T75 flask in the presence of 20 ml of DMEM high glucose + 10% heat decomplemented low endotoxin FBS. After 6 days, 20 ml of L-929 conditioned medium/T75 flask were collected, filtered (0.2 µm) and stored at −20 °C. Macrophage differentiation medium (DMEM high glucose + 10% HIS-LE FBS + 10% L-929 CM) was replaced at the third day and at the fifth day of the differentiation process. 6 days after launching the differentiation, macrophages were detached by trypsinization and by the use of a cell scraper and were then seeded at 750,000 cells/6-well (Greiner) for M0 and at 500,000 cells/well for M1 and M2, in macrophage differentiation medium. 24 h after seeding, murine macrophages were used as M0 or were polarized for 24 h in M1 with 10 ng/ml LPS (#L8630, Sigma) and 20 ng/ml rmIFNγ (R&D Systems) or for 24 h in M2 with 20 ng/ml rmIL-4 (R&D Systems) and 20 ng/ml rmIL-13 (R&D Systems).

### Chronic and cycling hypoxia exposure

Macrophages were incubated in CO_2_ independent medium supplemented with 4 mM L-glutamine (Sigma) and 3.75 g/l D-glucose (Merck). Normoxic cells (N) were incubated in the same conditions but in normal atmosphere (21% O_2_). For chronic hypoxia (chH), cells were exposed to a continued period of 6 h under 1% O_2_. For cycling hypoxia (cyH), cells were exposed to four consecutive cycles of 1 h hypoxia (1% O_2_) followed by 30 min reoxygenation (air, 21% O_2_) (6 h). In order to expose cells to hypoxia, a homemade pressurized incubator was used. N_2_ gas was injected (and air contained in the incubator was rejected) into this incubator until the 1% O_2_ 99% N_2_ concentration was reached. The O_2_ concentration was measured with an Eppendorf electrode.

### NF-κB-pathway and JNK inhibition

The NF-κB-pathway inhibitor (Bay11-7082; S2913, Selleckchem) or JNK inhibitor (SP600125; Sigma Aldrich) were added to the THP-1 macrophages at 10 μM or 30 μM 1 h or 2 h before the hypoxia experiments in the CO_2_ independent medium, respectively. Then, the macrophages were exposed to N, chH or cyH during 6 h. The efficiency of the inhibition of p65 nuclear translocation was analyzed by immunofluorescence and the efficiency of the phosphorylation of c-jun inhibition was confirmed by western blotting (Supplementary Figs. 1A and 2A). The absence of toxicity of Bay11-7082 and SP600125 were confirmed by western blotting for cleaved PARP and MTT assay, respectively (Supplementary Figs. 1B and 2B).

### Immunofluorescence labeling

Immunofluorescence labeling was performed as described before^21,22^. Briefly, cells were fixed 10 min in 4% paraformaldehyde in PBS. Cells were washed with PBS, then permeabilized with 0.1% Triton X100 in PBS during 5 min. Cells were blocked with 2% BSA in PBS 30 min and incubated O/N with primary antibody at 4 °C (CST #8242; p65; 1:400 diluted in PBS BSA 2%). Cells were rinsed 30 min in PBS BSA 2% and incubated with secondary antibody (Alexa Fluor 488-conjugated anti-rabbit IgG antibody; Molecular Probes, #A11034). Cells were then incubated with TOPRO-3 to stain the nucleus. The coverslips were mounted on Mowiol (Sigma) and the pictures taken with confocal microscope (SP5, Leica).

### MTT assay

150 000 THP-1 cells were differentiated in macrophages in 24-well plate as described before. Cells were incubated 8 h with SP600125 at 10, 20, 50 or 100 μM in 500 μL of CO_2_ independent medium during 8 h. Then, 500 μL of MTT solution (2.5 mg/mL in PBS; Sigma; #M2128) were added and cells were incubated 2 h at 37 °C. Media were removed and cells were incubated 1 h at 37 °C in 1 ml of lysis Buffer ((SDS 30%/ N,N-dimethyl-formamide 2:1 pH 4.7), with 70 rpm agitation. Absorbance was then measured at 570 nm.

### RT-qPCR

After the incubation, total RNA was extracted from cells using the QIAcube system with RNeasy Mini kit (THP-1) or Micro kit (BMDM) and DNase digest protocol (QIAGEN). mRNA in 2 µg of total RNA was reverse transcribed by using Transcriptor First Strand cDNA synthesis kit (#4379012001, Roche). A sample processed without reverse transcriptase enzyme was used as negative control for qPCR analyses. The sequences of qPCR forward and reverse primers are available in Supplementary Table S1. Amplification reaction assays contained SYBRGreen PCR Master Mix (#4309155, Applied Biosystem) and primers (IDT, 300 nM). RPS9 was used as the reference gene for normalization and mRNA abundance was quantified using the threshold cycle method.

### ELISA

For cytokine secretion analysis, human or murine macrophages were seeded and polarized in 24-well plates at 150,000 cells/well. For N, chH, cyH incubation, 750 µl of CO_2_ independent medium + 4 mM L-glutamine + 3.75 g/l D-glucose were added per well. For murine BMDM, incubation medium was supplemented with 5% L-929 conditioned-medium. Cytokine concentrations in conditioned media were assayed using specific ELISA kits (Quantikine, R&D Systems) according to supplier’s recommendations. Cytokine concentrations (pg/ml) were normalized by total protein concentrations (µg/ml) determined by the Folin Method after cell lysis with 200 µl 0.5 N NaOH/well.

### Western blot analysis

Total protein extraction from macrophages plated in 6-well plates was performed using a lysis buffer containing 40 mM Tris pH 7.5, 150 mM KCl, 1 mM EDTA, 1% Triton-X-100, PIC (Protease Inhibitor Cocktail, Roche), PIB 25x (Phosphatase Inhibitor Buffer, 25 mM Na_3_VO_4_, 250 mM PNPP, 250 mM β-glycerophosphate, 125 mM NaF). Cell lysate was recovered and centrifuged for 5 min at 15,700 g and 4 °C to pellet cell debris. The supernatant was collected and stored at −70 °C before western blotting. 20 µg of proteins were separated on 10% SDS-PAGE gels and transferred onto a low fluorescence background PVDF blotting membrane (Millipore). Quantitative LI-COR technology was used for western blot analyses (Odyssey Infrared Imaging System v3.0.16, LI-COR, Biosciences). Membranes were blocked with Odyssey blocking buffer diluted 1:2 in PBS for 1 h at RT. Primary antibodies diluted in Odyssey Blocking buffer-Tween 0.1% were incubated overnight at 4 °C, then membranes were washed with PBS-Tween 0.1%, and finally incubated with secondary antibodies diluted 10,000 x for 1 h at RT. Membranes were washed with PBS-Tween 0.1%, and then with PBS, and finally dried before scanning. Loading control was assessed with α-tubulin or β-actin according to the molecular weight of the protein of interest. Antibodies used are listed in Supplementary Table S2.

### Statistical analysis

Data are reported as mean ± 1 SEM. Statistical analyses were performed using SigmaPlot Software. When normality tests failed, statistical analyses were performed on square root- or log-transformed data. Corresponding statistical tests are outlined in figure captions.

## Results

### Cycling hypoxia induces a pro-inflammatory phenotype in human M0 macrophages and amplifies the pro-inflammatory phenotype displayed by human M1 macrophages

**Figure 1:**
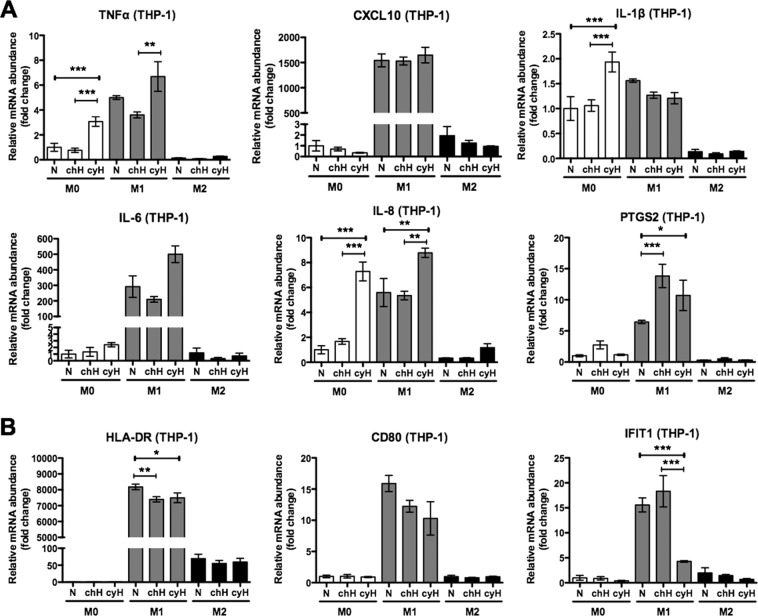
Effects of cycling hypoxia on the mRNA expression of M1 markers in human M0, M1 and M2 macrophages. THP-1 M0, M1 and M2 macrophages were exposed to normoxia (N), chronic hypoxia (chH) or cycling hypoxia (cyH) for 6 h. mRNA expression of pro-inflammatory (**A**) and intracellular host defense response (**B**) M1 markers was evaluated directly after the incubation by RT-qPCR (n = 3, mean ± 1 SEM). Statistical analysis was performed by two-way ANOVA and Holm-Sidak test as post hoc test. *P < 0.05; **P < 0.01; ***P < 0.001.

We firstly examined the impact of 4 cycles of 1 h hypoxia/30 min reoxygenation (cyH) on the mRNA expression of M1 and M2 markers in human THP-1 macrophages either unpolarized (M0) or polarized into M1 or M2 phenotype. The effects of cyH were compared to chronic hypoxia (chH) or normoxia (N). We separated the study of M1 markers in two categories with those contributing to inflammation and those playing a role in the intracellular host defense response. For pro-inflammatory M1 markers (Fig. 1A), in M0 macrophages, cyH significantly increased the mRNA expression of TNFα, IL-1β and IL-8. In M1 macrophages, cyH increased TNFα, IL-8 and PTGS2 mRNA expression. M2 macrophages were less affected by cyH compared to M0 and M1 macrophages, as only an increase in IL-8 mRNA expression was observed. For intracellular host defense response M1 markers (Fig. 1B), chH and cyH decreased HLA-DR (antigen presentation to T cells^23^) and CD80 (T cell activation^24^) mRNA expression in M1 macrophages, and cyH did not affect their expression neither in M0 nor in M2 macrophages. Moreover, cyH (but not chH) highly decreased IFIT1 (inhibition of viral replication^25^) mRNA expression in M1 macrophages while it left unaltered its expression in M0 or M2 macrophages. Regarding M2 markers (Supplementary Fig. 3), cyH did not alter their mRNA expression in M2 macrophages and did not induce their expression neither in M0 nor in M1 macrophages.

**Figure 2:**
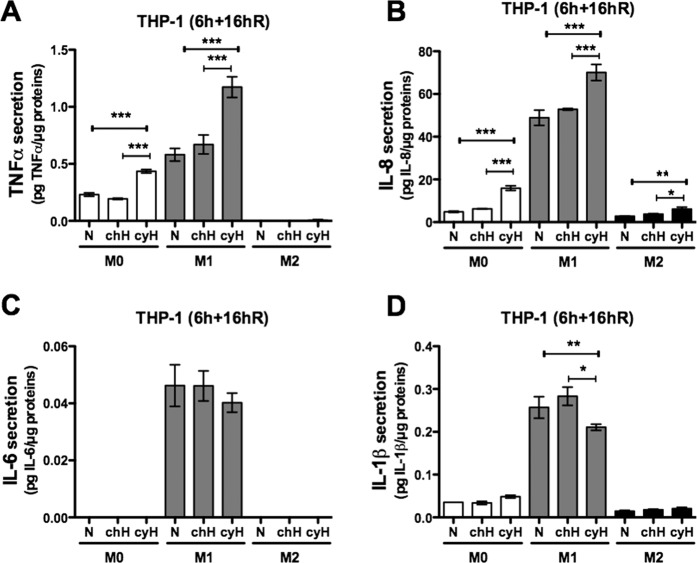
Effects of cycling hypoxia on the secretion of pro-inflammatory cytokines by human THP-1 M0, M1 and M2 macrophages. THP-1 M0, M1 and M2 macrophages were exposed to normoxia (N), chronic hypoxia (chH) or cycling hypoxia (cyH) for 6 h. Conditioned media were harvested 16 h of reoxygenation after the 6 h of incubation (6 h + 16hR). Concentration of TNFα (**A**), IL-8 (**B**), IL-6 (**C**) and IL-1β (**D**) was assayed by ELISA (n = 3, mean ± 1 SEM). Statistical analysis was performed by two-way ANOVA and Holm-Sidak test as post hoc test. *P < 0.05; **P < 0.01; ***P < 0.001.

In order to check whether these cyH–induced mRNA expression changes could lead to an effective pro-inflammatory phenotype induced in M0 macrophages or amplified in M1 macrophages, we investigated the secretion of pro-inflammatory cytokines. Cytokine concentrations were determined by ELISA after 16 h reoxygenation following the 6 h incubation under N, chH or cyH (6 h + 16hR) (Fig. 2). Firstly, it has to be noted that in comparison to M0 macrophages, M1 macrophages secreted higher amounts of TNFα, IL-8, IL-6 and IL-1β while M2 macrophages secreted lesser amounts of IL-8 and IL-1β and did not secrete TNFα and IL-6. cyH highly increased the secretion of TNFα by M0 macrophages to nearly reach the level detected for M1 macrophages under normoxia (N) (Fig. 2A). It also increased TNFα secretion by M1 macrophages. Moreover, cyH highly increased IL-8 secretion by all types of macrophages (Fig. 2B). In contrast, cyH did not increase IL-6 secretion by M1 macrophages (Fig. 2C) and significantly decreased IL-1β secretion by M1 macrophages (Fig. 2D).

Then, we examined whether a concomitant cyH exposure at the beginning of THP-1 macrophage polarization process could modulate the intensity of M1 and M2 polarization (Supplementary Fig. 4). Therefore, M0 macrophages were simultaneously exposed to 6 h of N, chH or cyH and to M1 or M2 polarization molecules and the polarization protocol was ended as usual before measurements of mRNA expression for M1 (Supplementary Fig. 4A,B) and M2 markers (Supplementary Fig. 4C). Results showed that this co-stimulation increased not only the mRNA expression of TNFα and PTGS2 in M1 macrophages but also significantly decreased the mRNA expression of CD206 and CCL22 in M2 macrophages, indicating that cyH is also able to partially impair the M2 polarization process.

### Cycling hypoxia induces a pro-inflammatory phenotype in murine M0 macrophages and amplifies the pro-inflammatory phenotype displayed by murine M1 macrophages

In contrast to THP-1 macrophages for which the protocol of polarization was previously well optimized by our team^22^, M1 and M2 polarization of murine bone marrow derived macrophages (BMDM) required fine tuning^26^. For M1 polarization, M0 macrophages were incubated for 24 h with LPS (1 ng/ml or 10 ng/ml) in combination with 20 ng/ml IFNγ. For M2 polarization, M0 macrophages were incubated with 20 ng/ml IL-4 and 20 ng/ml IL-13 for either 24 h or 48 h. M0 macrophages were placed in the same medium as the one used for M1 and M2 but without the polarization cocktail, and were used as control cells for polarization validation. We then evaluated the mRNA expression of M1 and M2 markers (Supplementary Fig. 5). In M1 polarized macrophages but not in M2, we observed an increase in the mRNA expression of the six pro-inflammatory cytokine coding genes (Supplementary Fig. 5A), of the three pro-inflammatory enzyme coding genes (Supplementary Fig. 5B) and of the three intracellular host defense response genes (Supplementary Fig. 5C). Of note, all M1 marker gene transcripts in M1 polarized macrophages reached higher expression levels in the presence of 10 ng/ml LPS (vs. 1 ng/ml). As this concentration is not cytotoxic for murine BMDM (data not shown), we used 10 ng/ml LPS in combination with 20 ng/ml IFNγ for M1 polarization in the next experiments. In M2 polarized macrophages, we observed an increase in the mRNA expression of MRC-1 and Arg-1 at both times of polarization but to a higher extent after 24 h (vs. 48 h) (Supplementary Fig. 5D). The 24 h timing was thus chosen for further experiments of M2 polarization from BMDM.

**Figure 3:**
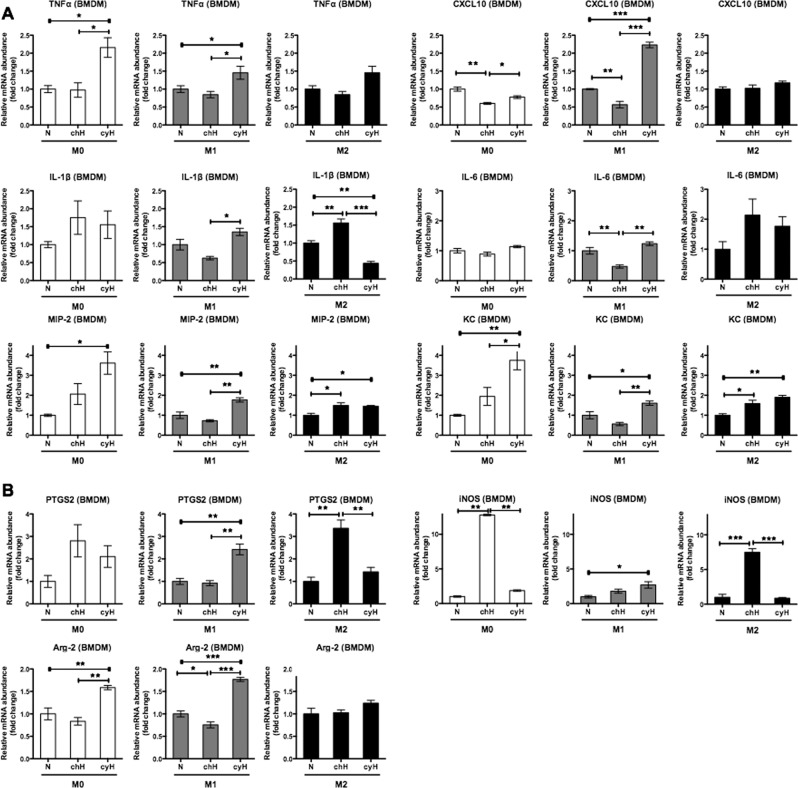
Effects of cycling hypoxia on the mRNA expression of M1 markers in murine M0, M1 and M2 macrophages. M0, M1 and M2 macrophages (BMDM) were exposed to normoxia (N), chronic hypoxia (chH) or cycling hypoxia (cyH) for 6 h. mRNA expression of pro-inflammatory cytokines (**A**) and pro-inflammatory enzymes (**B**) was evaluated directly after the incubation by RT-qPCR (n = 3, mean ± 1 SEM). Statistical analysis was performed by two-way ANOVA and Holm-Sidak test as post hoc test. *P < 0.05; **P < 0.01; ***P < 0.001.

After validating M1 and M2 polarization, we studied the effects of cyH on the mRNA expression of polarization markers in murine BMDM. The effects of cyH for M0, M1 and M2 macrophages are presented on different graphs. For the pro-inflammatory cytokines (Fig. 3A), cyH exerted a higher effect on M0 macrophages compared to M1 and M2 macrophages as we observed a highly significant increase in TNFα, MIP-2 and KC mRNA expression. In M1 macrophages, an increase in MIP-2 and KC mRNA expression was detected but to a lower extent than in M0 macrophages. However, a high increase in CXCL10 mRNA expression by cyH in M1 macrophages was evidenced. In M2 macrophages, cyH only increased MIP-2 and KC mRNA expression.

For the pro-inflammatory enzymes (Fig. 3B), cyH increased the mRNA expression of genes encoding the enzymes PTGS2, iNOS and Arg-2 in M1 macrophages. Moreover, a high increase in iNOS mRNA level was induced by chronic hypoxia in M0 and M2 macrophages. cyH increased Arg-2 mRNA expression in M0 and in M1 macrophages. cyH had no effect on the mRNA expression of these enzymes in M2 macrophages.

Regarding the intracellular host defense response markers (Supplementary Fig. 6), cyH decreased MARCO and IFIT1 mRNA expression in M0 macrophages but increased IFIT1 mRNA expression in M1 macrophages.

Then, we studied the effects of cyH on the mRNA expression of the two M2 markers (Supplementary Fig. 7). CyH slightly induced the mRNA expression of Arg-1 in M2 macrophages and induced the mRNA expression of MRC-1 in M0 macrophages. Interestingly, chH increased Arg-1 mRNA expression in M1 and M2 macrophages and, to a very high extent, in M0 macrophages.

**Figure 4:**
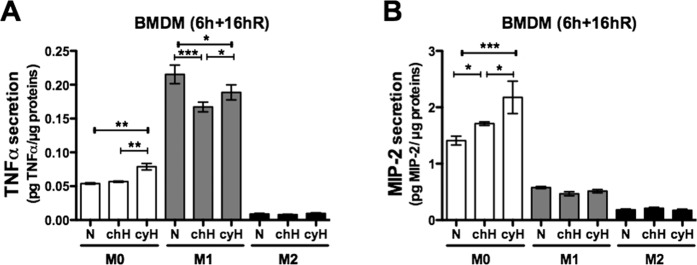
Effects of cycling hypoxia on the secretion of pro-inflammatory cytokines by murine macrophages. M0, M1 and M2 macrophages were exposed to normoxia (N), chronic hypoxia (chH) or cycling hypoxia (cyH) for 6 h. Conditioned media were harvested 16 h of reoxygenation after the 6 h of incubation (6 h + 16hR). Concentration of TNFα (**A**) and MIP-2 (**B**) was assayed by ELISA (n = 3, mean ± 1 SEM). Statistical analysis was performed by two-way ANOVA and Holm-Sidak test as post hoc test. *P < 0.05; **P < 0.01; ***P < 0.001.

Then, we investigated the effects of cyH on the secretion of pro-inflammatory cytokines. Therefore, murine M0, M1 and M2 BMDM were exposed for 6 h to N, chH or cyH and then conditioned media were harvested after 16 h reoxygenation (6 h + 16hR). Results showed that M1 macrophages secreted more TNFα than M0 macrophages while M2 macrophages secreted less TNFα. cyH increased TNFα secretion by M0 macrophages but not by M1 macrophages (Fig. 4A). M0 macrophages secreted much higher amounts of MIP-2 than M1 and M2, and MIP-2 secretion by M0 macrophages was increased when exposed to cyH (Fig. 4B).

### Cycling hypoxia differentially activates STAT1, NF-κB and c-jun transcription factors in macrophages as function of polarization and tissue origin

Since most of the effects of cyH were related to an enhancement of the inflammation associated to the M1 phenotype, we next analyzed the effects of cyH on the major transcription factors described to be involved in M1 polarization, namely STAT1, NF-κB, AP-1 and IRF5^27^. To this aim, we studied, by western blotting, the phosphorylated (active) forms of STAT1, p65 (one of the subunits of NF-κB) and c-jun (one of the subunits of AP-1). For IRF-5, we studied the total abundance of the protein (and not a post-translational modification) as variations in the total abundance directly reflect stimulation.

**Figure 5:**
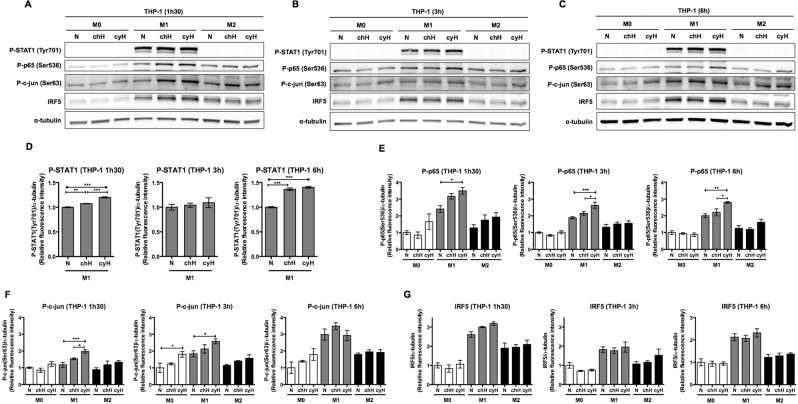
Effects of cycling hypoxia on the abundance of the phosphorylated form of STAT1, p65 and c-jun as well as on the abundance of IRF5 in human M0, M1 and M2 macrophages. THP-1 M0, M1 and M2 macrophages were exposed to normoxia (N), chronic hypoxia (chH) or cycling hypoxia (cyH) for 1h30 (**A**), for 3 h (**B**) or for 6 h (**C**), and then total protein extraction was performed. Abundance of the phosphorylated form of STAT1 (Tyr701), p65 (Ser536) and c-jun (Ser63) as well as the total abundance of IRF5 was detected by western blotting (n = 3). α-tubulin was used as loading control. Fluorescence intensity of each immunoblotted protein was quantified and normalized for α-tubulin (**D–G**). Statistical analysis was performed by two-way ANOVA (or one-way ANOVA for P-STAT1 in M1 macrophages) and Holm-Sidak test as post hoc test. *P < 0.05; **P < 0.01; ***P < 0.001.

For human THP-1 macrophages (Fig. 5), total protein extracts were recovered from M0, M1 and M2 macrophages exposed to N, chH or cyH after 1h30 (A), 3 h (B) and 6 h (C). Results showed that phosphorylated STAT1 (Tyr701) was only detected in M1 macrophages and that the level of phosphorylated p65 (Ser536), phosphorylated c-jun (Ser63) and total IRF5 was higher in M1 than in M0 and in M2 macrophages. In M0 macrophages, cyH increased the abundance of P-c-jun (Fig. 5F). In M1 macrophages, cyH increased the abundance of P-p65 (Fig. 5E), of P-c-jun (Fig. 5F) and to a lesser extent, of P-STAT1 (Fig. 5D). In M2 macrophages, a trend to an increase in the abundance of P-p65 and P-c-jun was observed in response to cyH.

**Figure 6:**
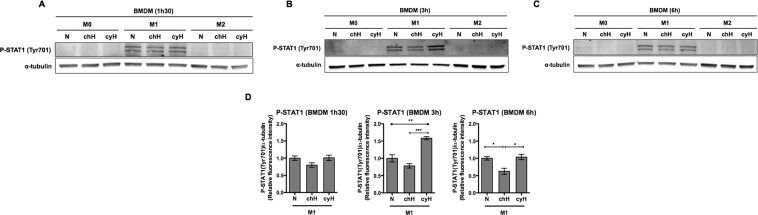
Effects of cycling hypoxia on the abundance of the phosphorylated form of STAT1 in murine M0, M1 and M2 macrophages. M0, M1 and M2 macrophages (BMDM) were exposed to normoxia (N), chronic hypoxia (chH) or cycling hypoxia (cyH) for 1h30 (**A**), for 3 h (**B**) or for 6 h (**C**), and then total protein extraction was performed. Abundance of the phosphorylated form of STAT1 (Tyr701) was detected by western blotting (n = 3). α-tubulin was used as loading control. Fluorescence intensity of the phosphorylated form of STAT1 was quantified and normalized for α-tubulin (**D**). Statistical analysis was performed by one-way ANOVA and Holm-Sidak test as post hoc test. *P < 0.05; **P < 0.01; ***P < 0.001.

For murine BMDM (Fig. 6 and Supplementary Fig. 8), total protein extracts were recovered from M0, M1 and M2 after 1h30 (A), 3 h (B) and 6 h (C). Phosphorylated STAT1 was only present in M1 macrophages and the level of phosphorylated p65, phosphorylated c-jun and IRF5 was higher in M1 macrophages compared to M0 or M2 macrophages. In M0, M1 and M2 macrophages, no significant change in the level of the phosphorylated form of p65, c-jun nor in the total abundance of IRF5 was observed under cyH in comparison to N (Supplementary Fig. 8). In contrast, in M1 macrophages, cyH increased the abundance of P-STAT1 (Fig. 6D) while chH significantly decreased it.

### NF-κB and c-jun activation is implicated in the induction by cycling hypoxia of a pro-inflammatory phenotype in human M0 macrophages and in the amplification of the pro-inflammatory phenotype in human M1 macrophages

**Figure 7:**
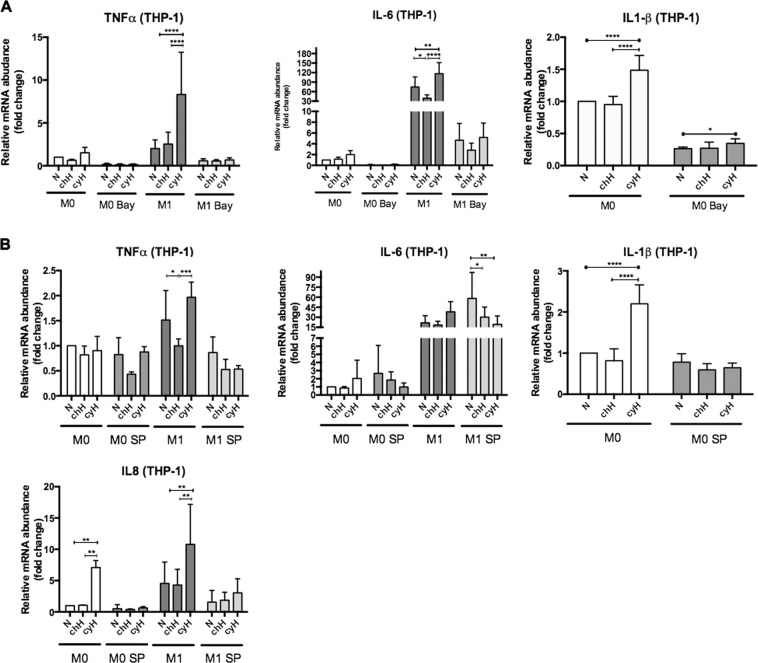
Effects of NF-𝛋B and c-jun inhibition on cycling hypoxia induced pro-inflammatory phenotype in human THP-1 M0 and M1 macrophages. THP-1 M0 and M1 macrophages were incubated with Bay11-7082 (10 μM) or SP600125 (30 μM) for 1 h or 2 h, respectively. Then, THP-1 M0 and M1 macrophages were exposed to normoxia (N), chronic hypoxia (chH) or cycling hypoxia (cyH) for 6 h. The mRNA expression of TNFα, IL-6, IL1-β and IL-8 was analyzed by RT-qPCR (n = 4, mean ± 1 SEM). Statistical analysis was performed by two-way ANOVA and Holm-Sidak test as post hoc test. *P < 0.05; **P < 0.01; ***P < 0.001; ****P < 0.0001.

In order to examine whether the effects of cyH on THP-1 M0 and M1 macrophages were due to NF-κB or c-jun activation, M0 and M1 macrophages were pre-incubated with the NF-κB-pathway inhibitor Bay11-7082 or with the JNK inhibitor SP600125 before exposure to N, chH or cyH. The efficiency and inocuity of NF-κB-pathway and JNK inhibitors were validated (Supplementary Figs. 1 and 2). The increase in TNFα and IL-6 expression induced by cyH in M1 macrophages was completely abolished by the inhibition of NF-κB or c-jun (Fig. 7A,B). Furthermore, the increased expression of IL-8 induced by cyH were slightly inhibited by SP600125 in M1 macrophages and abolished in M0 macrophages. The increase in IL-1β expression in M0 macrophages induced by cyH was partly inhibited in the presence of Bay11-7082 and totally abolished in the presence of SP600125 (Fig. 7A,B). These results indicate that NF-κB and c-jun are implicated in the pro-inflammatory effects induced by cyH in THP-1 M0 and M1 macrophages.

### Cycling hypoxia induced a c-jun/p65 signaling pathway in THP-1 M0 and M1 macrophages

**Figure 8:**
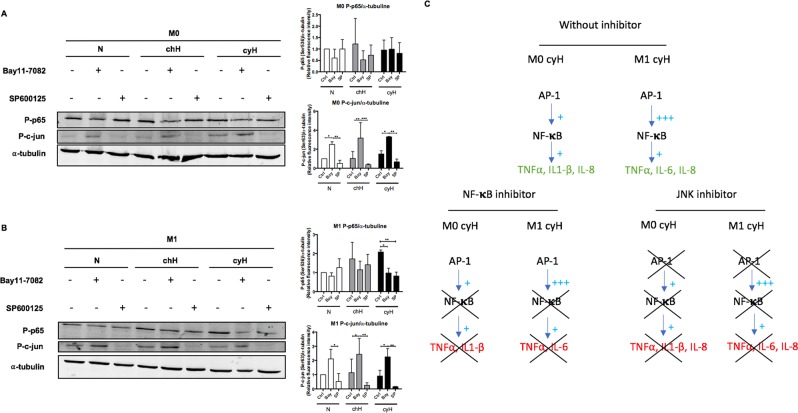
Effects of NF-𝛋B and c-jun inhibition on the protein abundance of the phosphorylated form of p65 and c-jun. THP-1 M0 and M1 macrophages were incubated with Bay11-7082 (10 μM) or SP600125 (30 μM) for 1 h or 2 h, respectively. Then, these THP-1 M0 (**A**) and M1 (**B**) macrophages were exposed to normoxia (N), chronic hypoxia (chH) or cycling hypoxia (cyH) for 1 h 30 min (M1) or 4 h 30 min (M0). Abundance of the phosphorylated form of p65 (Ser536) and c-jun (Ser63) were detected by western blotting (n = 3). Fluorescence intensity of each immunoblotted protein was quantified and normalized for α-tubulin. Statistical analysis was performed by two-way ANOVA and Holm-Sidak test as post hoc test. *P < 0.05; **P < 0.01; ***P < 0.001.The results are summarized in (**C**).

Because p65 and c-jun inhibition were both able to abolish the pro-inflammatory effects of cyH on THP-1 M0 and M1 macrophages, we wondered if the activation of p65 could influence the activation of c-jun, and vice-versa. M0 and M1 THP-1 macrophages were incubated with Bay11-7082 or SP600125 under N, chH or cyH during 1h30 (M1) or 4h30 (M0). The phosphorylation of p65 and c-jun was then analyzed by western blotting (Fig. 8). As expected, the phosphorylation of p65 and c-jun was inhibited in M0 and M1 macrophages by Bay11-7082 and SP600125, respectively. In M0 macrophages, JNK inhibition did not influence p65 phosphorylation level (Fig. 8A). In M1 macrophages, JNK inhibition decreased p65 phosphorylation in macrophages exposed to cyH and to a lesser extent to macrophages exposed to N or chH (Fig. 8B). In both types of macrophages, the phosphorylation of c-jun was strongly increased by Bay11-7082. All these results are summarized and indicated that a c-jun/p65 signaling pathway is activated in M0 and M1 macrophages exposed to cyH (Fig. 8C).

## Discussion

Inflammation is one of the hallmarks of cancer associated with bad prognosis^12^. Previously, we showed that cyH enhanced the pro-inflammatory phenotype of endothelial cells *in vitro* and enhanced tumor inflammation *in vivo*^10^. Numerous studies have evidenced the critical role of immune cells in tumor progression and inflammation. TAMs display a remarkable plasticity to environmental cues. Indeed, in the early state of tumor, TAMs display mostly monocyte/M1 phenotype, whereas at later stages, TAMs mostly belong to the M2 phenotype^28–30^. While M1 macrophages are pro-inflammatory, they have anti-tumoral effects, associated with cytotoxicity towards cancer cells, and immune-stimulatory functions^30^. On the other hand, while M2 macrophages are anti-inflammatory, they have pro-tumoral effects due to immune-suppression and angiogenesis induction^31^. Since TAMs are key cells involved in the control of tumor inflammation and progression, we investigated the role of cyH on the phenotype of human and murine M0, M1 and M2 macrophages.

In human THP-1 M0 macrophages, cyH promoted a pro-inflammatory phenotype characterized by an increase in TNFα and IL-8 secretion but did not induce the functions related to intracellular host defense. CyH thus favors a shift of unpolarized M0 macrophages towards a M1-like phenotype that is incomplete and possibly not fully functional. In M1 macrophages, cyH amplified the pro-inflammatory phenotype evidenced by an increase in TNFα and IL-8 secretion but partially decreased the expression of intracellular host defense genes, HLA-DR, CD80 and IFIT1. Interestingly, the decrease in the expression of the intracellular host defense genes has already been observed in a mouse tumor model of sleep apnea, a phenomenon associated with intermittent hypoxia^32^. Furthermore, hypoxia has already been described to inhibit the ability of macrophages for phagocytosis and for T cell activation via the reduction of CD80 expression^33^. Moreover, cyH increased the mRNA expression of PTGS2, the gene encoding cyclooxygenase 2 (COX2). COX2-derived PGE2 is known to inhibit the functions of T cells^34,35^ and infiltration of COX2-expressing macrophages is associated with tumor neovascularization and tumor growth^36^. Altogether our data suggest that in response to cyH, THP-1 M1 macrophages reveal a decreased potential to trigger an adaptive immune response toward cancer cells together with a stimulation of angiogenesis.

In murine BMDM, cyH also induced a pro-inflammatory phenotype in unpolarized M0 macrophages characterized by an enhanced secretion of TNFα and MIP-2, and decreased the basal level of the intracellular host defense response. In murine M1 macrophages, cyH amplified the pro-inflammatory phenotype especially by increasing the expression of pro-inflammatory enzyme genes while it did not alter the intracellular host defense response. The major effects of cyH evidenced in murine macrophages were observed in M0 macrophages. Unlike THP-1 M1 macrophages, murine M1 macrophages did not appear to be highly sensitive to cyH. This could be explained by the higher concentration of LPS used for the murine M1 polarization.

Our results demonstrated that M1-associated genes were not all induced or amplified by cyH, suggesting that cyH specifically modulated some aspects of the phenotype of macrophages. This modulation depends on transcription factors activated under cyH exposure. We focused on the major transcription factors described to orientate the M1 polarization. In murine BMDM, cyH mainly increased STAT1 activation in M1 macrophages. In THP-1 macrophages, cyH increased c-jun activation in M0 and M1 macrophages. In addition, it highly increased NF-κB activation and to a lesser extent STAT1 activation in M1 macrophages. No significant changes were observed in human M2 macrophages. Interestingly, we showed that p65 and JNK inhibition in M0 and M1 macrophages were both able to inhibit the pro-inflammatory phenotype induced by cyH (Fig. 7). Interestingly, JNK inhibition decreased slightly the phosphorylation of p65 in M1 macrophages exposed to N or chH and to a higher extent in M1 macrophages exposed to cyH. JNK inhibition did not changed p65 phosphorylation level in M0 macrophages (Fig. 8). Furthermore, p65 inhibitor decreased p65 phosphorylation and increased c-jun phosphorylation, which means that c-jun is unable to induce by itself and independently of NF-κB, the cyH-induced pro-inflammatory phenotype in M0 and M1 macrophages. Taken together, these results support a model wherein cyH-induced or amplified a pro-inflammatory phenotype in M0 and M1 macrophages via a c-jun/p65 signaling pathway (Summarized in Fig. 8C).

In our *in vitro* experimental model, cyH was performed by 4 cycles of 1-hour hypoxia followed by 30 minute reoxygenation. This protocol was based on *in vivo* measurements of pO_2_ fluctuations in the tumor vasculature occurring at the frequency of 0.5 to 3 cycles per hour^9,37^. Furthermore, the O_2_ saturation in tumor is comprised between 1 to 2% O_2_ in a majority of solid tumors^38^. It was showed *in vitro* that 1-hour hypoxia causes a rapid accumulation of HIF-1α, whereas 30-minute reoxygenation is sufficient to abrogate this accumulation^39^. Moreover, a progressive accumulation of HIF-1α along cycles was observed in endothelial cells^40,41^. This *in vitro* protocol was used to demonstrate that cyH increased endothelial cell migration, tubulogenesis and endothelial cell resistance towards proapoptotic stresses, and increased tumor cell radioresistance^39,42,43^. More recently, we demonstrated that this timing of cyH amplified the TNFα-induced pro-inflammatory state of endothelial cells since an increase in both pro-inflammatory cytokine secretion and endothelial monocyte adhesion was observed^10^.

In order to study the effects of obstructive sleep apnea (OSA), Murphy *et al*. showed that hypoxia/reoxygenation cycles can induce a pro-inflammatory phenotype to THP-1 M0 and M1 macrophages. The protocol of hypoxia/reoxygenation used was not relevant to cancer research. Indeed, extremely rapid changes in O_2_ saturation only 8 h a day for 3 consecutive days (40 s 16% O_2_, 40 s 3% O_2_) were performed. Schaefer *et al*. showed that hypoxia/reoxygenation cycles (6 cycles of 40 min 1% O_2_ 20 min 21% O_2_) induces a pro-inflammatory phenotype in THP-1 M0 macrophages characterized by an increased expression of pro-inflammatory cytokine such as TNFα, IL-6 and IL-1β. In order to see the effects of OSA on the development of atherosclerosis, Zhou *et al*. showed that hypoxia/reoxygenation cycles (6 cycles of 35 min 0.1% or 5% O_2,_ followed by 25 min N) induced a pro-inflammatory phenotype in unpolarized M0 THP-1 macrophages. The pO_2_ saturation used in these several studies during cyH was either too low or too high for cancer research, since the O_2_ saturation in tumor is comprised between 1 to 2% O_2_ in a majority of solid tumors^38^. In these conditions, they showed that the advanced glycation end-products (AGE) receptor (RAGE) was implicated in the cyH pro-inflammatory effects. Some ligands of RAGE, namely AGE and HMGB1, were also observed to induce pro-inflammatory phenotype in M0 macrophages and in human bronchial epithelial cells, respectively^44,45^. Hence, it would be interesting to study the effects of cyH in conditions relevant to cancer research on the expression and secretion of such RAGE ligands by macrophages and if there exists a crosstalk between c-jun/p65 and RAGE.

Some limitations in the study can be highlighted. The first one is the pO_2_ used in the study. Indeed, in human healthy tissue, the physiological normoxia is comprised mostly between 4% O_2_ (muscle) and 9.5% O_2_ (kidney, outer cortex)^46,47^. In this study, normoxia and the cyH reoxygenation were performed by exposing cells to atmospheric air (21% O_2_). Nonetheless, the hypoxia value that we used was physiologically relevant since O_2_ saturation in tumor is comprised between 1 and 2% O_2_ in a majority of solid tumors^38,47^. Secondly, we showed that cyH induced a pro-inflammatory phenotype in M0 and M1 macrophages in both BMDM and THP-1 macrophages. If there are some similarities between these two types of macrophages, we also observed some differences notably in fold induction and cytokine expression and secretion. Furthermore, the pro-inflammatory response was dependent in NF-κB and c-jun activation in THP-1 macrophages whereas cyH induced mostly STAT1 activation. The discrepancy between murine and human macrophages was well characterized in^48^. Indeed, Spiller *et al*. compared human macrophages (either derived from peripheral blood or from induced pluripotent stem cells either from THP-1 monocytes) to BMDM. Up to 800 genes were used to characterize the expression profile of each model. It was shown that human macrophages were more closely related to each other than to mouse macrophages. This could explain the differences of cyH effects between human and murine macrophages observed in our study. However, Spiller *et al*. also observed some discrepancies between human peripheral blood (PB)-derived macrophages and THP-1 macrophages^48^. Thereby, it would be interesting to confirm the effects of cyH on human PB-derived macrophages. Nonetheless, THP-1 macrophages model is a very good and reliable model which is commonly used in the literature^49^. THP-1 macrophages are more stable and show less heterogeneity in comparison to PB-derived macrophages. The difference between THP-1 macrophages and human monocyte-derived macrophages is much smaller than that of between THP-1 monocytes and human monocytes^7^. Furthermore, Shiratori *et al*. compared THP-1 macrophages and human macrophages in terms of phagocytic capacity and M1 and M2 polarization. They showed that THP-1 macrophages was an appropriate model to study the M1 polarization but less for M2 polarization. In addition, no difference in phagocytic capacity between the two models was observed. Finally, the response of THP-1 macrophages to LPS is very similar to PB-derived macrophages^50^.

In conclusion, in this study, we are the first to investigate the effects of cyH and chH comparatively in human and murine macrophages and in M0, M1 and M2 macrophages. We investigated for the first time the effects of cyH on the activation of transcription factors involved in M1 polarization and on the secretory phenotype of human and murine macrophages. We demonstrated that cyH, on its own, induces or amplifies a pro-inflammatory phenotype in M0 and M1 macrophages, but not in M2 macrophages. These phenotypes were characterized by an increase in the secretion of pro-inflammatory cytokines and driven by a c-jun/p65 signaling pathway. These effects were specific to cyH, since they were not observed in cells exposed to chH. Thereby, the present work highlights the role of cyH in the amplification of inflammation, in agreement with our previous results emphasizing for the first time the link between cyH and tumor inflammation^10^. Indeed, here we demonstrated that cyH induces a pro-inflammatory phenotype in macrophages characterized by an increase in TNFα secretion, while we previously revealed that cyH amplifies the TNFα-induced inflammatory response of endothelial cells characterized by an increase in pro-inflammatory cytokine secretion and in monocyte adhesion^10^. This means that an amplification loop of the effects of cyH on tumor inflammation exists, as TNFα secreted in higher amount by cycling hypoxic macrophages could target endothelial cells and as cyH amplifies the endothelial inflammatory response to TNFα. This enhanced local inflammation could provoke an increase in blood vessel permeabilization and thus could favor cancer cell intravasation and dissemination. This could be an explanation by which cyH increases tumor metastasis^51–54^.

Altogether, these results evidenced a global mechanism initiated specifically by cyH, that could account for the amplification of tumor-promoting inflammation. CyH induces a common mechanism in different cell types of the tumor microenvironment, leading to the establishment of an inflammatory microenvironment. Understanding the molecular mechanism involved in the cyH-induced effects could allow to highlight original therapeutic targets with the advantage to inhibit a global mechanism supporting tumor progression without affecting healthy tissue.

## Supplementary information

Supplementary Data
