# Supplementary material for: Cycling hypoxia promotes a pro-inflammatory phenotype in macrophages via JNK/p65 signaling pathway
Source: Sci Rep. 2020 Jan 21;10:882. doi: 10.1038/s41598-020-57677-5 (PMC6972721; doi:10.1038/s41598-020-57677-5)
Supplement: Supplementary file 1 — Supplementary Data [file 41598_2020_57677_MOESM1_ESM.pdf]

Cycling hypoxia promotes a pro-inflammatory phenotype in macrophages via JNK/p65 signaling pathway

Victor DELPRAT, Céline TELLIER, Catherine DEMAZY, Martine RAES, Olivier FERON, Carine MICHIELS

Supp Table S1

**qPCR primers for human genes**			
RPS9	F : CTGGATGAGGGCAAGATGAAG<br>R : GTCTGCAGGCGTCTCTCTAAGAA	HLA-DRα	F: CATAAGTGGAGTCCCTGTGCTA<br>R: TCAGGATTCAGATAGAACTCGGC
TNFα	F : CTGCACTTTGGAGTGATCGG<br>R : TCAGCTTGAGGGTTTGCTAC	CD80	F : ACGCCCTGTATAACAGTGTCC<br>R : GAGGAAGTTCCCAGAAGAGGTC
CXCL10	F : AAGTGGCATTCAAGGAGTACC<br>R : ATGCAGGTACAGCGTACAGT	IFIT1	F: CCTCCTTGGGTTCGTCTACA<br>R: TTCTCAAAGTCAGCAGCCAGT
IL-1β	F : GCCCTAAACAGATGAAGTGCTC<br>R : GAGATTCGTAGCTGGATGCC	Fibronectin	F : TGTGGTTGCCTTGCACGAT<br>R : GCTTGTGGGTGTGACCTGAGT
IL-6	F : CCTGAACCTTCCAAAGATGGC<br>R : CACCAGGCAAGTCTCCTCATT	CD206	F : GCTAAACCTACTCATGAATTACTTACAACAA<br>R : GAAGACGGTTTAGAAGGGTCCAT
IL-8	F : TCTGTGTGAAGGTGCAGTTTT<br>R : GGGGTGGAAAGGTTTGGAGTA	CCL22	F : TGTGGTTGCCTTGCACGAT<br>R : GCTTGTGGGTGTGACCTGAGT
PTGS2	F : ATTAGCCTGAATGTGCCATAAGACT<br>R : ACCCACAGTGCTTGACACAGAAT		
**qPCR primers for mouse genes**			
RPS9	F: GCTGTTGACGCTAGACGAGA<br>R: AGCATTGCCTTCAAACAGACG	iNOS	F: CAATGGCAACATCAGGTCGG<br>R: CGTACCGGATGAGCTGTGAA
TNFα	F: GAACTTCGGGGTGATCGGT<br>R: CTCCTCCACTTGGTGGTTTG	Arg-2	F: GAGACCACAGCCTGGCAATAG<br>R: ATGTCCGCATGAGCATCAAC
CXCL10	F: GAAATCATCCCTGCGAGCCTA<br>R: ATCGTGGCAATGATCTCAACA	MARCO	F: ACTCCAGAGGGAGAGCACTT<br>R: TTGTCCAGCCAGATGTTCCC
IL-1β	F: TGCCACCTTTTGACAGTGATG<br>R: ATGTGCTGCTGCGAGATTTG	CD80	F: ACAGTCGTCGTCATCGTTGT<br>R: CCCGAAGGTAAGGCTGTTGT
IL-6	F: CTCTGCAAGAGACTTCCATCC<br>R: TGAAGTCTCCTCTCCGGACT	IFIT1	F: ACAGCTACCACCTTTACAGCAA<br>R: TGAAGCAGATTCTCCATGACCT
MIP-2	F: CGCCCAGACAGAAGTCATAG<br>R: TCCTCCTTTCCAGGTCAGTTA	Arg-1	F: GTACATTGGCTTGCGAGACG<br>R: TTTCTTCCTTCCCAGCAGGT
KC	F: GCAGACCATGGCTGGGATT<br>R: CCTGAGGGCAACACCTTCAA	MRC-1	F: GGATTGCCCTGAACAGCAAC<br>R: ACTTAAGCTTCGGCTCGTCA
PTGS2	F: AGCAGATGACTGCCCAACTC<br>R: GGGTCAGGGATGAACTCTCTC		

Supplementary Table S1. List of qPCR primers (5’-3’)

Supp Table S2

**Primary antibodies**		
Reference	Incubation time	Dilution
Rabbit mAb anti-phospho-STAT1 (Tyr701) (H+M reactivity), Cell signaling, 58D6, #9167	O/N 4°C	1/1 000
Rabbit mAb anti-phospho-p65 (Ser536) (H+M reactivity), Cell signaling, 93H1, #3033	O/N 4°C	1/1 000
Rabbit polyclonal Ab anti-phospho-c-jun (Ser63) (H+M reactivity), Cell signaling, #9261	O/N 4°C	1/1 000
Mouse mAb anti-IRF5 (EPR17067) (H+M reactivity), Abcam, #ab181553	1 h RT	1/2 000
Mouse mAb anti-PARP (H reactivity), BD Pharmingen, #551024	O/N 4°C	1/2 000
Mouse mAb anti-α-tubulin (H+M reactivity), sigma, #T5168	45 min RT	1/20 000
Mouse mAb anti-β-actin (AC-15), Sigma, #A5441	30 min RT	1/10 000
**Secondary antibodies**		
Reference	Incubation time	Dilution
Goat anti-mouse IgG IRDye conjugated, LI-COR, Biosciences, #926-69070 (680), #926-32210 (800)	1 h RT	1/10 000
Goat anti-rabbit IgG IRDye conjugated, LI-COR, Biosciences, #926-68071 (680), #926-32211 (800)	1 h RT	1/10 000

Supplementary Table S2. References of primary and secondary antibodies used for Western blot analyses.

Supp Fig.1

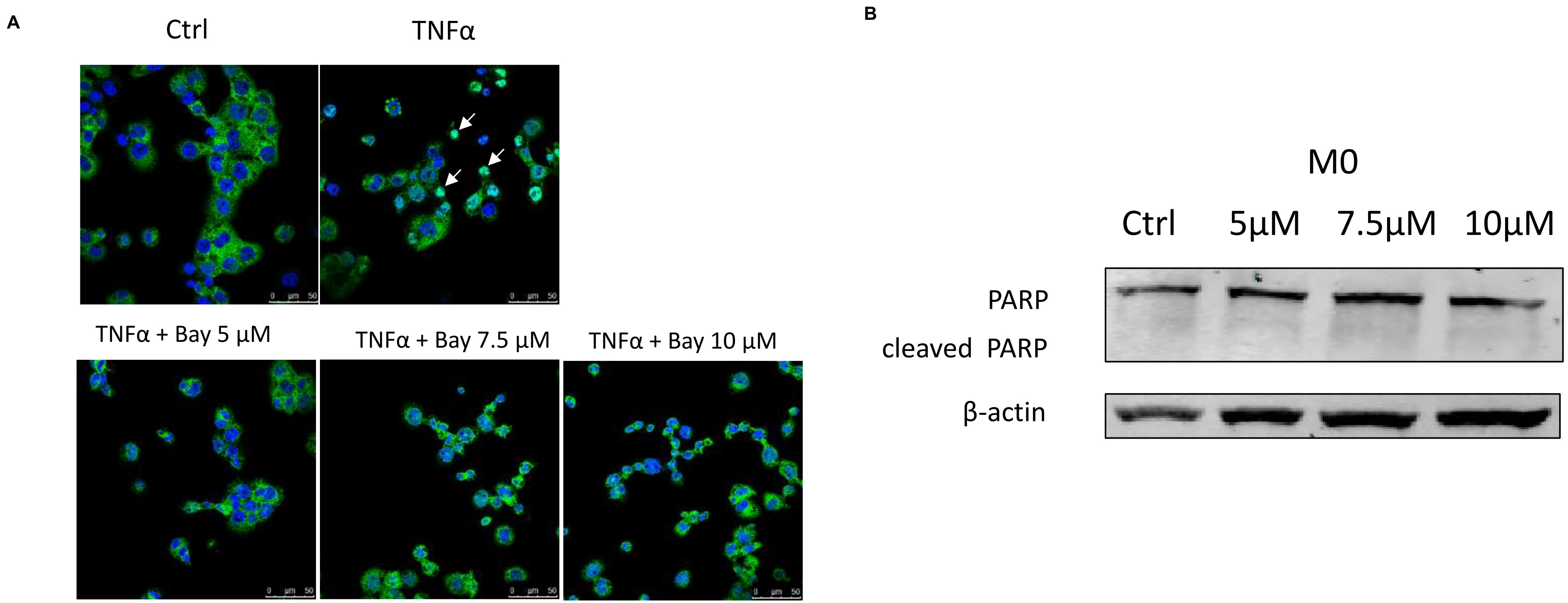

Supplementary Figure 1. Bay11-7082 efficiency and toxicity. (A) THP-1 M0 macrophages were incubated with Bay11-7082 at 5, 7.5 and 10 µM during 1h. Then, cells were treated 30 min with 20 ng/ml of TNFα. Immunofluoresnce staining of p65 was then analyzed to measure the effects of Bay11-7082 on p65 translocation into the nucleus (white arrows ; n=1). (B) THP-1 M0 macrophages were incubated with Bay11-7082 at 5, 7.5 or 10 µM during 7h. Then, the total abundance of PARP and cleaved PARP was analyzed by western blotting (n=1).

Supp Fig.2

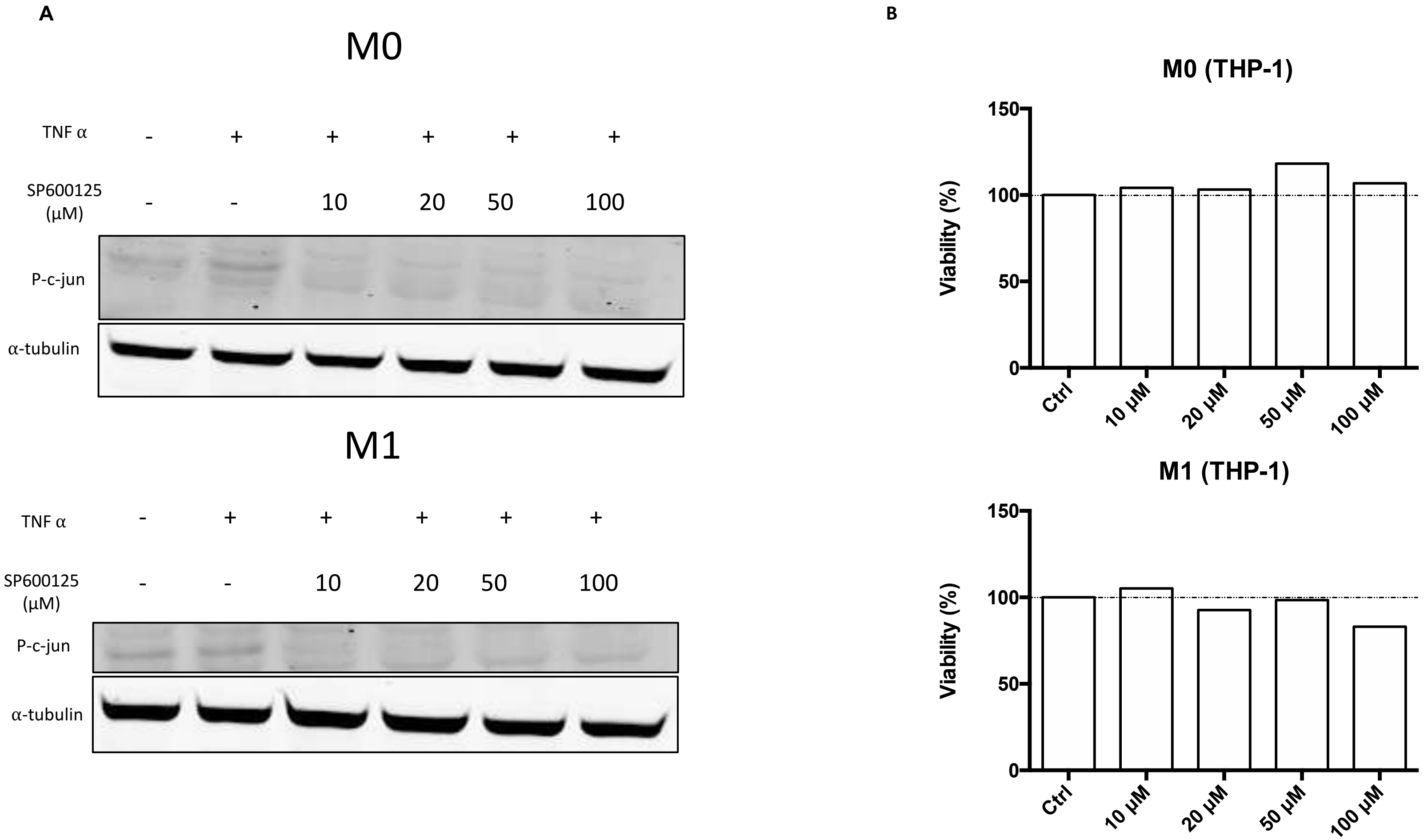

Supplementary Figure 2. SP600125 efficiency and toxicity. (A) THP-1 M0 and M1 macrophages were incubated with SP600125 at 10, 20, 50 or 100 µM during 2h. Then, cells were treated 45 min with 20 ng/ml of TNFα. The abundance of P-c-jun was then analyzed by western blotting to measure the efficiency of SP600125 to inhibit the phosphorylation of c-jun (n=1). (B) THP-1 M0 and M1 macrophages were incubated with SP600125 at 10, 20, 50 or 100 µM during 8h. Then, the viability of cells was analyzed by MTT assay (n=1).

Supp Fig.3

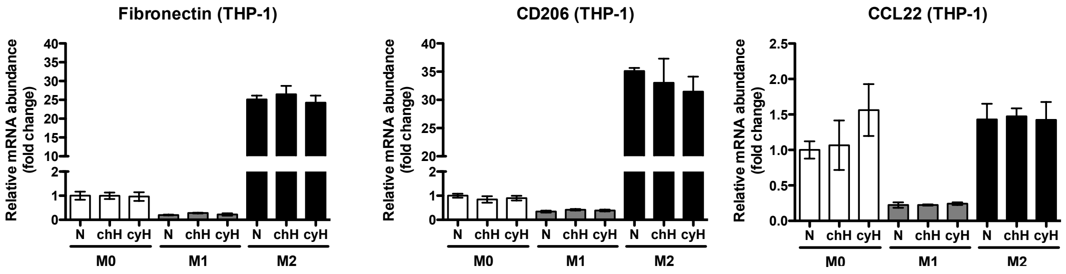

Supplementary Figure 3. Effects of cycling hypoxia on the mRNA expression of M2 markers in human M0, M1 and M2 macrophages. THP-1 M0, M1 and M2 macrophages were exposed to normoxia (N), chronic hypoxia (chH) or cycling hypoxia (cyH) for 6 h. mRNA expression of M2 markers was evaluated directly after the incubation by RT-qPCR (n=3, mean ± 1 SEM). Statistical analysis was performed by two-way ANOVA and Holm-Sidak test as post hoc test.

Supp Fig.4

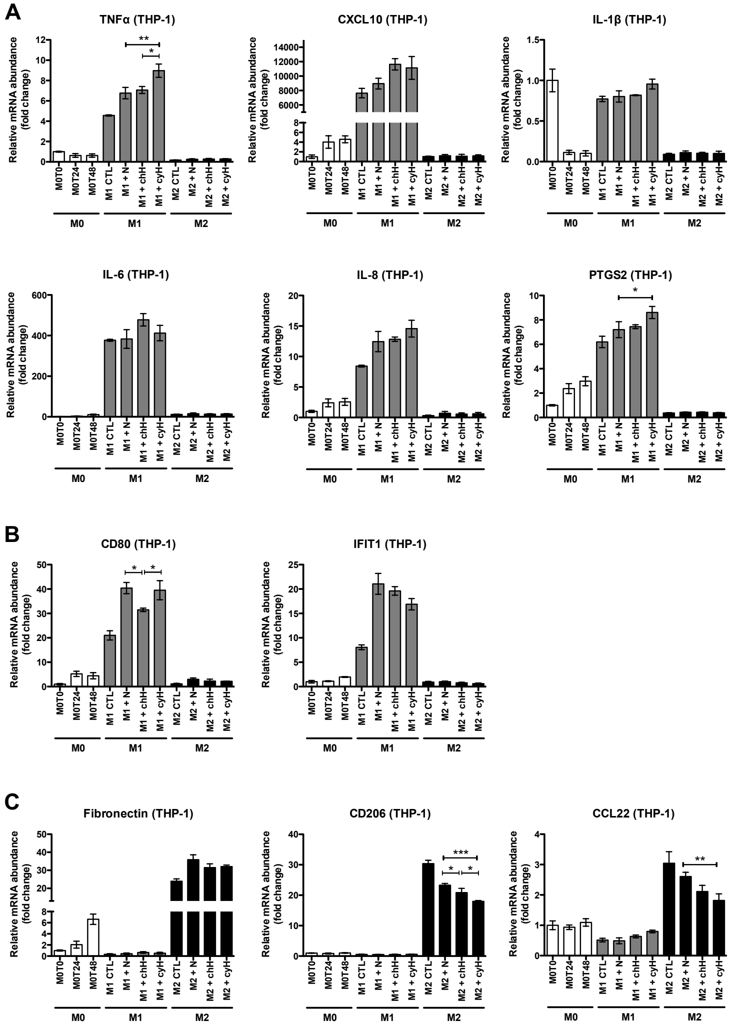

Supplementary figure 4. Modulation of the mRNA expression of M1 and M2 markers by a simultaneous exposure of human macrophages to cycling hypoxia and polarization molecules. THP-1 M0 macrophages were exposed to normoxia (N), chronic hypoxia (chH) or cycling hypoxia (cyH) for 6 h simultaneously with the beginning of M1 or M2 polarization. After the 6 h of co-incubation, cell medium was replaced and M1 or M2 polarization stimulation was continued (+ 18 h for M1 and + 42 h for M2). mRNA expression of M1 markers (A, B) and M2 markers (C) was evaluated after the complete polarization (24 h for M1 and 48 h for M2) by RT-qPCR (n=3, mean ± 1 SEM). Statistical analysis was performed by two-way ANOVA and Holm-Sidak test as post hoc test. *, $P < 0.05$; **, $P < 0.01$; ***, $P < 0.001$

Supp Fig.5

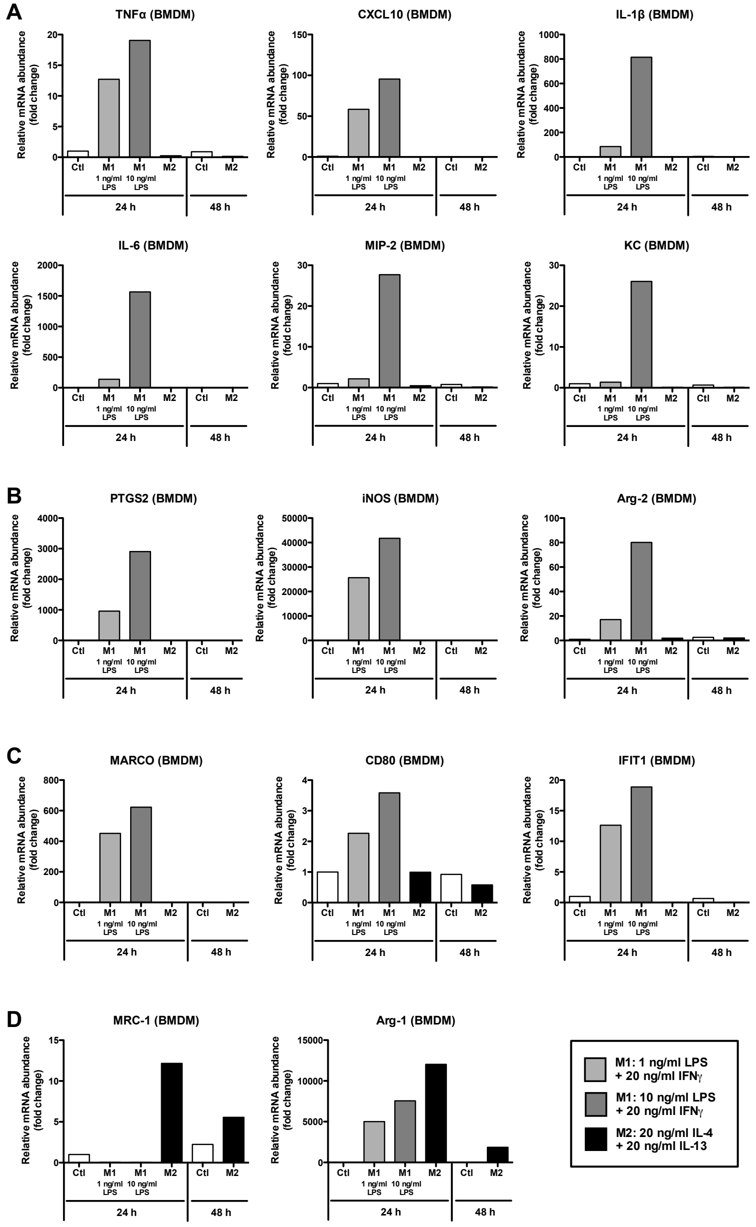

Supplementary Figure 5. Validation of M1 and M2 polarization of murine bone marrow-derived macrophages (BMDM). For M1 polarization, BMDM were incubated with IFNγ (20 ng/ml) and LPS (either 1 or 10 ng/ml) for 24 h. For M2 polarization, BMDM were incubated with IL-4 (20 ng/ml) and IL-13 (20 ng/ml) either for 24 h or for 48 h. Control BMDM (Ctl) were kept in the cell culture medium without polarization molecules for 24h or for 48 h. mRNA expression of M1 markers (A, B, C) and M2 markers (D) was evaluated by RT-qPCR (n=1).

Supp Fig.6

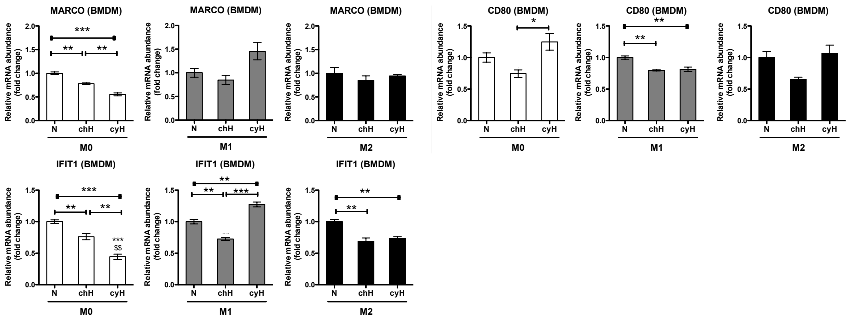

Supplementary Figure 6. Effects of cycling hypoxia on the mRNA expression of intracellular host defense markers in murine M0, M1 and M2 macrophages. M0, M1 and M2 macrophages (BMDM) were exposed to normoxia (N), chronic hypoxia (chH) or cycling hypoxia (cyH) for 6 h. mRNA expression of intracellular host defense markers was evaluated directly after the incubation by RT-qPCR (n=3, mean ± 1 SEM). Statistical analysis was performed by two-way ANOVA and Holm-Sidak test as post hoc test. *, $P < 0.05$; **, $P < 0.01$; ***, $P < 0.001$

Supp Fig.7

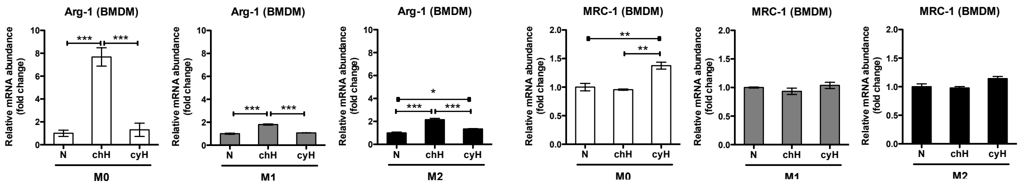

Supplementray Figure 7. Effects of cycling and chronic hypoxia on the mRNA expression of M2 markers in murine M0, M1 and M2 macrophages. M0, M1 and M2 macrophages (BMDM) were exposed to normoxia (N), chronic hypoxia (chH) or cycling hypoxia (cyH) for 6 h. mRNA expression of M2 markers was evaluated directly after the incubation by RT-qPCR (n=3, mean ± 1 SEM). Statistical analysis was performed by two-way ANOVA and Holm-Sidak test as post hoc test. *, $P < 0.05$; **, $P < 0.01$; ***, $P < 0.001$

Supp Fig.8

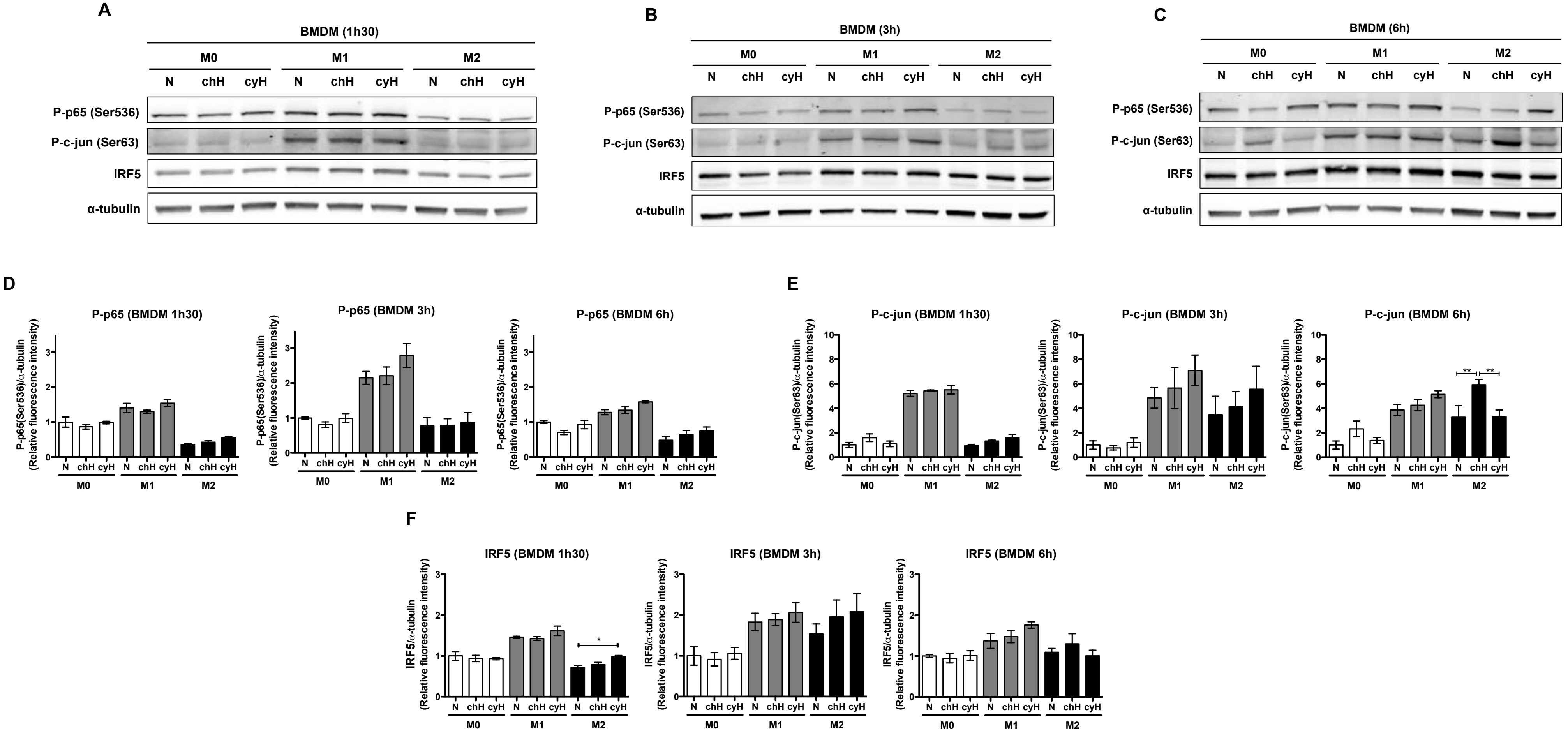

Supplementary Figure 8. Effects of cycling hypoxia on the abundance of the phosphorylated form of p65 and c-jun as well as on the abundance of IRF5 in murine M0, M1 and M2 macrophages. M0, M1 and M2 macrophages (BMDM) were exposed to normoxia (N), chronic hypoxia (chH) or cycling hypoxia (cyH) for 1h30 (A), for 3 h (B) or for 6 h (C), and then total protein extraction was performed. Abundance of the phosphorylated form of p65 (Ser536) and c-jun (Ser63) as well as the total abundance of IRF5 was detected by western blotting (n=3). α-tubulin was used as loading control. Fluorescence intensity of each immunoblotted protein was quantified and normalized for α-tubulin (D, E, F, G). Statistical analysis was performed by two-way ANOVA and Holm-Sidak test as post hoc test. *, $, #, $P < 0.05$; **, $$, ##, $P < 0.01$

Supp Fig.9

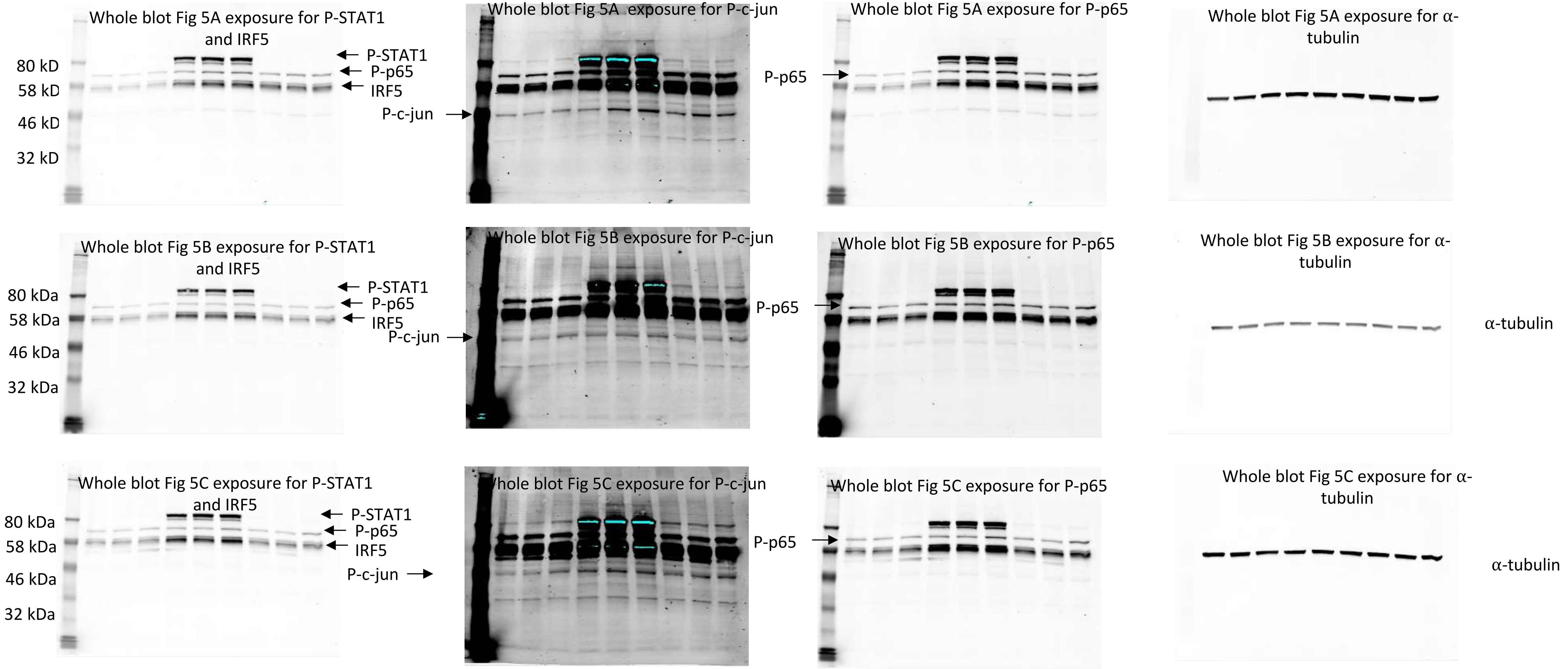

Supplementary Figure 9. Figure 5 whole blots, with the different exposure times used for each protein. The same membrane was incubated with antibodies against P-STAT1, IRF5, P-p65,P-c-jun and α-tubulin. We used red fluorescence for P-STAT1 , IRF5, P-p65, P-c-jun. We used green fluorescence for α-tubulin.

Supp Fig.10

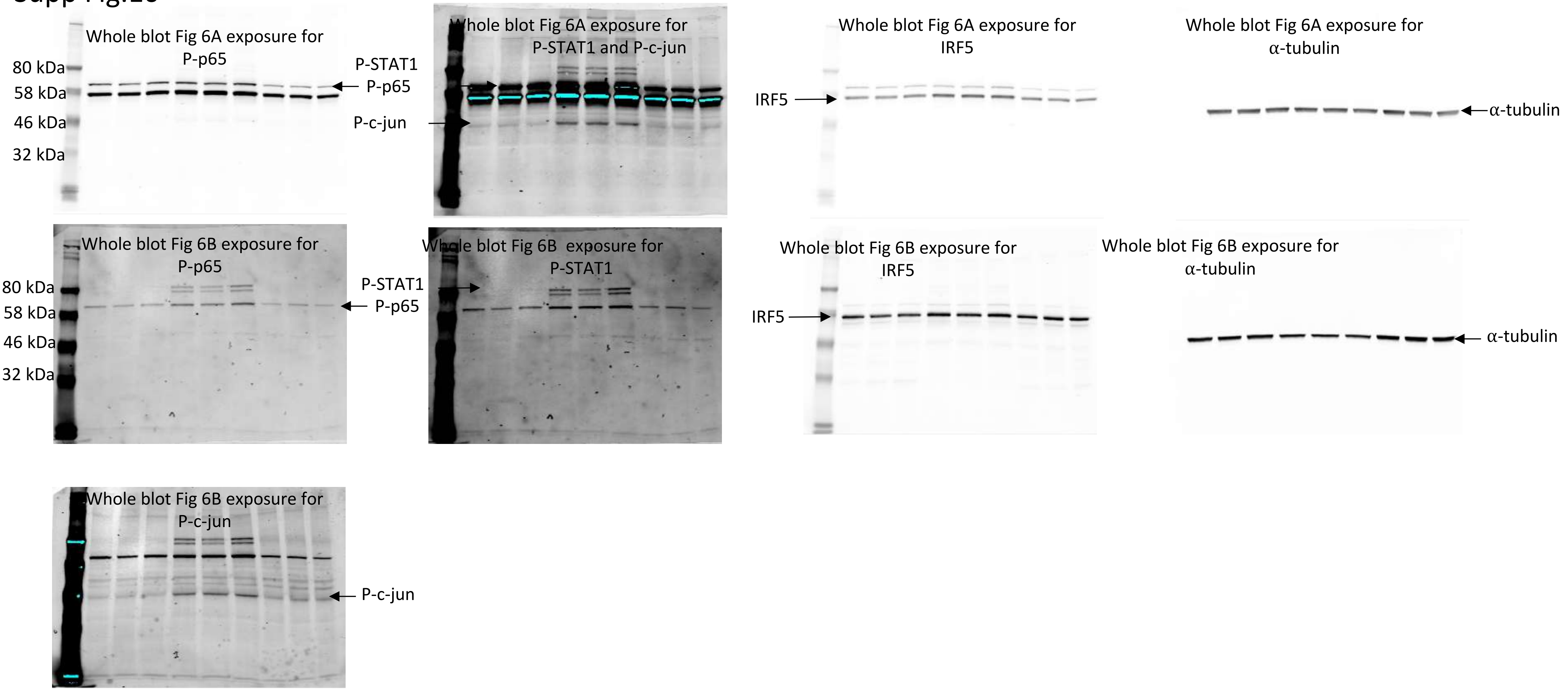
Whole blot Fig 6A exposure for P-p65
80 kDa
58 kDa
46 kDa
32 kDa
P-STAT1
P-p65
P-c-jun
Whole blot Fig 6A exposure for P-STAT1 and P-c-jun
Whole blot Fig 6A exposure for IRF5
IRF5
Whole blot Fig 6A exposure for α-tubulin
α-tubulin
Whole blot Fig 6B exposure for P-p65
80 kDa
58 kDa
46 kDa
32 kDa
P-STAT1
P-p65
Whole blot Fig 6B exposure for P-STAT1
Whole blot Fig 6B exposure for IRF5
IRF5
Whole blot Fig 6B exposure for α-tubulin
α-tubulin
Whole blot Fig 6B exposure for P-c-jun
P-c-jun

Supp Fig.10 (continued)

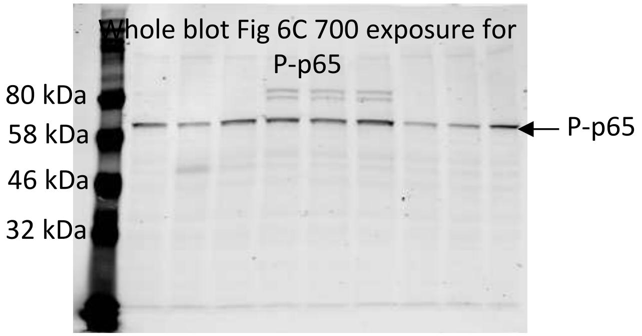

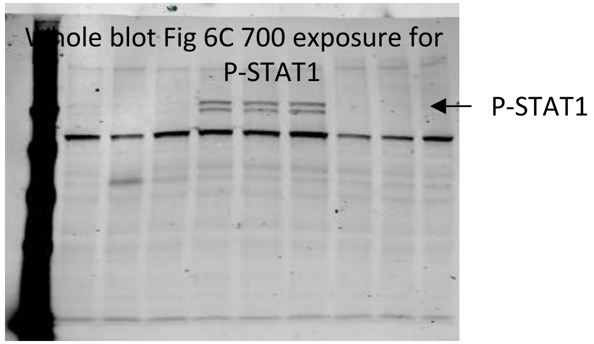

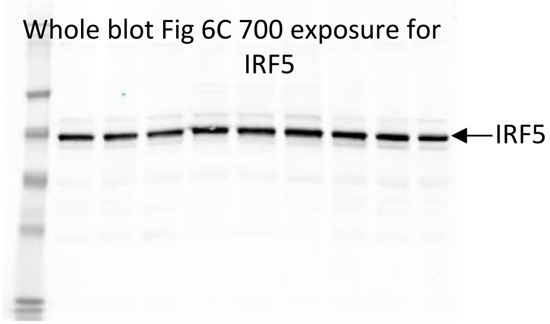

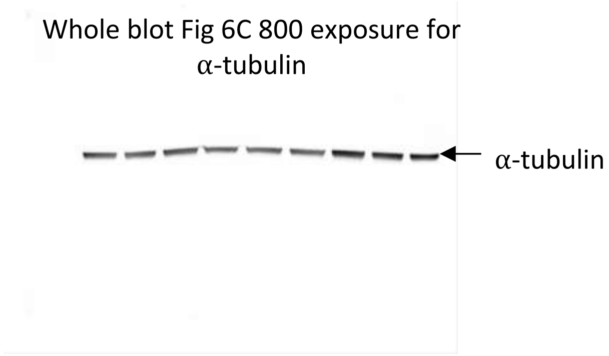

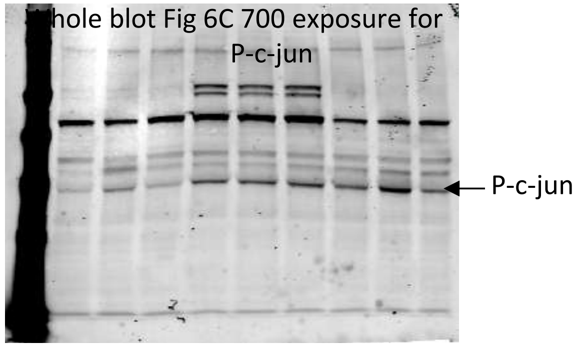

Supplementary Figure 10. Figure 6 whole blots, with the different exposure times used for each protein. The same membrane was incubated with antibodies against P-STAT1, IRF5, P-p65,P-c-jun and α-tubulin. We used red fluorescence for P-STAT1 , IRF5, P-p65, P-c-jun. We used green fluorescence for α-tubulin.

Supp Fig.11

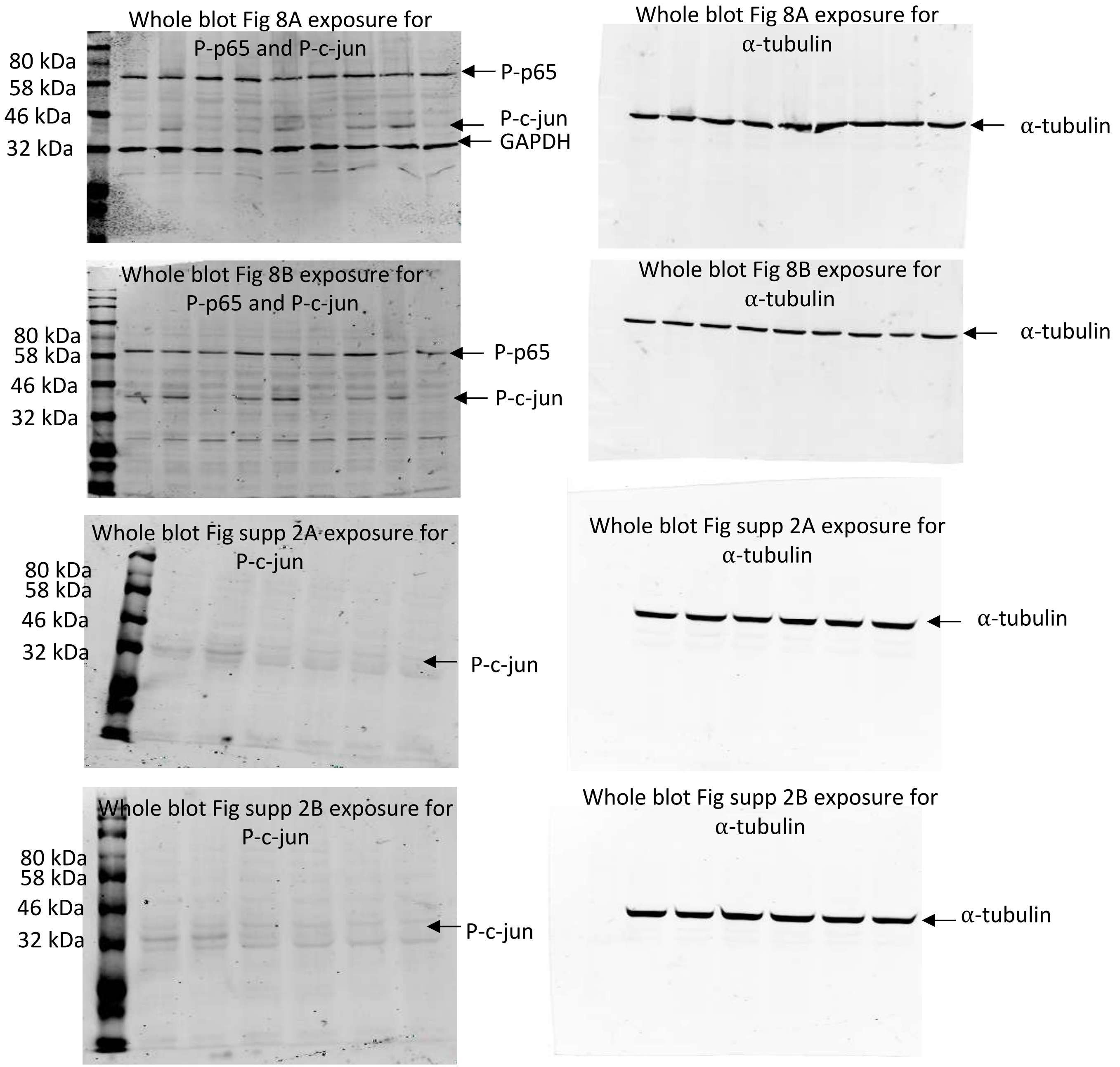

Supplementary Figure 11. Figure 8 and supplementary Fig 2B whole blots, with the different exposure times used for each protein. In figure 8, the same membrane was incubated with antibodies against P-p65, P-c-jun and α-tubulin. We used red fluorescence for P-p65 and P-c-jun and green fluorescence for α-tubulin. In Supplementary Fig 2B, the same membrane was incubated with antibodies against P-c-jun and α-tubulin. We used red fluorescence for P-c-jun and green fluorescence for α-tubulin.
